# The Influence of Luminophore Orientation and Dynamics on Circularly Polarised Luminescence (CPL) Spectroscopy

**DOI:** 10.1007/s10895-025-04563-w

**Published:** 2025-10-20

**Authors:** Lennart B. -Å. Johansson

**Affiliations:** Department of Chemistry, Umeå University, Umeå, S-90 187 Sweden

**Keywords:** Uniaxial anisotropy, Liquid crystals, Lipid bilayers, Birefringence

## Abstract

Circularly Polarised Luminescence (CPL) spectroscopy often deals with the generation of circularly polarised photons from macroscopically isotropic systems, such as liquid or solid solutions. The magnitude of CPL transitions depends on inherent specific properties of chiral chromophores/luminophores, which is connected to the strength and mutual intra-molecular orientation of the molecular electronic and magnetic transition dipoles. The present work aims to theoretically investigate the influence of orientation and dynamics on CPL emission for luminophores dissolved in microscopic and macroscopic anisotropic systems. Commonly studied systems of this kind are (liquid) crystals, lipid membranes, or model membranes. Typically, they exhibit uniaxial symmetry, and the luminophores considered may undergo fast, intermediate, or negligible reorientations on the timescale of luminescence emission. CPL from anisotropic and isotropic systems have been compared and the obtainable molecular information is described and discussed. Macroscopically isotropic systems, which are simultaneously microscopic anisotropic, are here exemplified by lipid vesicles. Taken together, a fundamental theoretical treatment is presented with connection to various experimental conditions. In brief, different experimental setups and aspects for monitoring the CPL emission are described, as well as commented on.

## Introduction

Circularly Polarised Luminescence (CPL) refers to the emission of fluorescent or phosphorescent left- and right-circularly polarised photons from chiral chromophores.

**Fig. 1:**
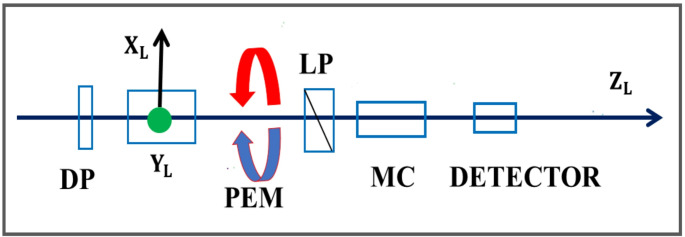
A typical schematic displaying the principal setup of a Circularly Polarised Luminescence spectrometer [2]. The excitation beam is usually polarised, but it is here chosen to be depolarised, DP. The sample is associated with a laboratory fixed Cartesian frame$\:{\:{(\mathrm{X}}_{\mathrm{L}},{\mathrm{Y}}_{\mathrm{L}},\mathrm{Z}}_{\mathrm{L}})$. The emitted radiation is passed through a photo-elastic modulator (PEM), a linear polariser (LP), a monochromator (MC) and analysed by means of the look-in amplifier technique

CPL originates from two consecutive one-photon events, firstly the excitation of a chromophore (i.e., absorption) followed by an emission process. The processes of CPL are separated in time due to the excited state lifetime of a luminophore. Luminophores dissolved in isotropic samples excited by a beam of light, initially comprise an anisotropic orientation distribution, as a result of photo-selection [1]. Exceptions are chromophores with inherent tetrahedral or higher symmetries. In a wider context, CPL experiments are analogous to common fluorescence depolarisation experiments [1]. In previous publications the setup of common CPL experiments have been described, see for instance [2, 3]. Figure 1 displays the fundamental experimental setup of a CPL spectrometer. The description involves a laboratory fixed frame ($\:{\mathrm{X}}_{\mathrm{L}},\:{\mathrm{Y}}_{\mathrm{L}},\:{\mathrm{Z}}_{\mathrm{L}})$, and a depolarised excitation beam impinging along a laboratory (L) $\:{\mathrm{Z}}_{\mathrm{L}}$-axis. The CPL emission is also detected along this axis after transmission through a photo-elastic modulator (PEM), that operates combined with a lock-in amplifier (Fig. 1). The PEM approach is most often applied in the construction of modern circular dichroism absorption spectrometers (see e.g [4].,, and papers cited therein).

**Fig. 2:**
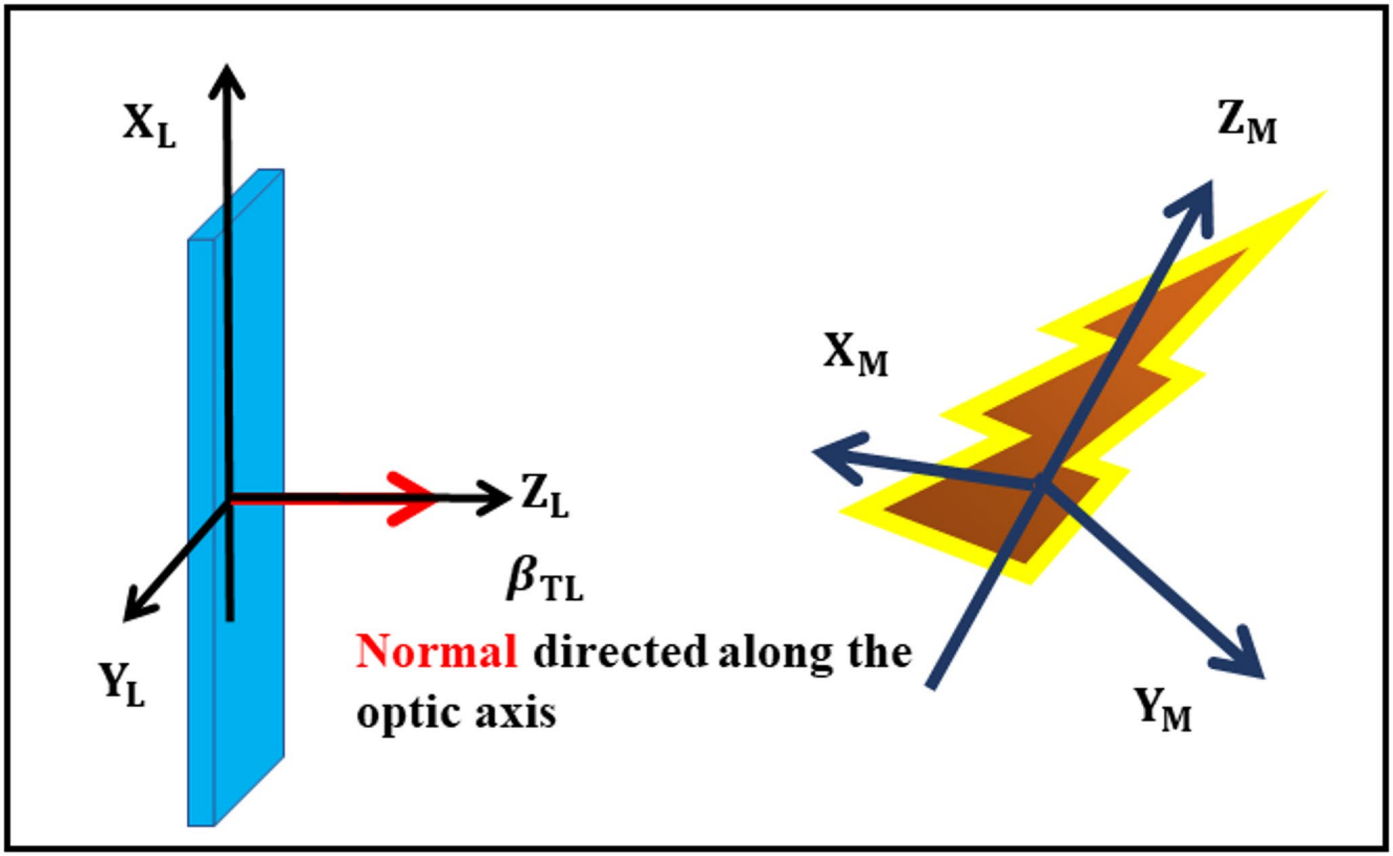
The left schematic exemplifies a laboratory fixed coordinate system $\:\left\{\mathrm{L}\:=\:{{(\mathrm{X}}_{\mathrm{L}}{,\mathrm{Y}}_{\mathrm{L}},\mathrm{Z}}_{\mathrm{L}})\right\}$ together with depolarised excitation light beam that propagates along the laboratory Z_L_-axis. The **N**ormal to a macroscopically oriented uniaxial sample (e.g., a lipid membrane) is colinear with the Z_L_-axis that coincides with the optic axis. To the right is schematically displayed a chiral chromophore and its connection to a molecule (M) fixed frame $\:\left\{\mathrm{M}\:=\:{{(\mathrm{X}}_{\mathrm{M}}{,\mathrm{Y}}_{\mathrm{M}},\mathrm{Z}}_{\mathrm{M}})\right\}$

A depolarised excitation field ensures a uniaxially symmetric orientation distribution of excited chromophores about the $\:{\mathrm{Z}}_{\mathrm{L}}$-axis. Consequently, the CPL emission is also uniaxially symmetric about this axis. For samples contained in e.g., standard fluorescence cuvettes (1 × 1 cm^2^), the depolarised excitation would be created along the $\:{\mathrm{Z}}_{\mathrm{L}}$-axis, by a beam propagating along the $\:{\mathrm{X}}_{\mathrm{L}}$-axis or $\:{\mathrm{Y}}_{\mathrm{L}}$-axis with a linear polarisation chosen to be along the$\:{\:\mathrm{Z}}_{\mathrm{L}}$-axis. For samples located in thin layers e.g., between quartz plates, the depolarised excitation is achieved by a depolarised beam propagating at normal incidence, i.e., along the propagation or the optic axis. Such an experimental arrangement is indicated in Fig. 2.

An important property of CPL spectroscopy is the emission dissymmetry factor ($\:{g}_{\mathrm{C}\mathrm{P}\mathrm{L}}$), which is defined as

1 $$\:{g}_{\mathrm{C}\mathrm{P}\mathrm{L}}\left(\lambda\:\right)=\frac{2{{\Delta\:}I}_{\mathrm{C}\mathrm{P}\mathrm{L}}\left(\lambda\:\right)}{{I}_{\mathrm{l}\mathrm{e}\mathrm{f}\mathrm{t}}\left(\lambda\:\right)+\:{I}_{\mathrm{r}\mathrm{i}\mathrm{g}\mathrm{h}\mathrm{t}}\left(\lambda\:\right)}\:$$

Here, $\:{I}_{\mathrm{l}\mathrm{e}\mathrm{f}\mathrm{t}}\left(\lambda\:\right)$ and $\:{I}_{\mathrm{r}\mathrm{i}\mathrm{g}\mathrm{h}\mathrm{t}}\left(\lambda\:\right)$ denote the separately measured left- and right-circularly polarised components of the emitted radiation (see for instance [3]). The observable,

2 $$\:{{\Delta\:}I}_{\mathrm{C}\mathrm{P}\mathrm{L}}\left(\lambda\:\right)=\:{I}_{\mathrm{l}\mathrm{e}\mathrm{f}\mathrm{t}}\left(\lambda\:\right)-{I}_{\mathrm{r}\mathrm{i}\mathrm{g}\mathrm{h}\mathrm{t}}\left(\lambda\:\right)$$

is commonly referred to as the emission circular intensity differential.

The present study, however, focuses on the influence of uniaxial macroscopic and microscopic orientation anisotropy on time-resolved, as well as, steady-state CPL experiments. Hence, the experiments may involve relaxation processes on nanosecond, microsecond, or slower time scales. Therefore, the time-resolved $\:{{\Delta\:}I}_{\mathrm{C}\mathrm{P}\mathrm{L}}(\lambda\:,t)$ is here emphasised in a somewhat different context than what is the common.

### Theoretical Prerequisites

The interaction between electromagnetic radiation and chromophores induces an electronic transition between an initial (0) and a final (f) state (0 $\:\to\:$ f). The process depends on the molecular electric and magnetic transition dipole moments, in what follows, denoted by $\:\overrightarrow{\mu\:}$ and $\:\overrightarrow{m}$, respectively. Most often, the probability of exciting a chromophore ($\:{P}_{0\:\to\:\:\mathrm{f}}$) is dominated by the dipole interaction between $\:\overrightarrow{\mu\:}$ and an electric field polarisation ($\:\overrightarrow{\varepsilon\:}$) according to $\:{P}_{0\:\to\:\:\mathrm{f}\:}\propto\:{\left|\overrightarrow{\mu\:}\cdot\:\overrightarrow{\varepsilon\:}\right|}^{2}$.

Throughout the present work, the starting moment is the excitation of luminophores by a short light pulse with a negligible half-width duration on the timescale of the luminescence lifetime ($\:\tau\:$). In practise, most chiral (bio)luminescent molecules exhibit an emission circular intensity differential, which typically corresponds to the detection of small intensity differences. Therefore, a slightly different formulation of Eq. 1 is introduced according to,

3 $$\:{{\Gamma\:}}_{\mathrm{C}\mathrm{P}\mathrm{L}}\left(\lambda\:\right)=\frac{{{\updelta\:}I}_{\mathrm{C}\mathrm{P}\mathrm{L}}(\lambda\:,t)}{{I}_{\mathrm{d}}(\lambda\:,t)}\:$$

where

4 $$\begin{aligned}{{\updelta\:}I}_{\mathrm{CPL}}\left(\lambda\:,t\right)&=\:\left\{{I}_{\mathrm{left}}\left(\lambda\:,t\right)-{I}_{\mathrm{right}}(\lambda\:,t)\right\}\\&={f}_{\mathrm{CPL}}\left(\lambda\:\right)\langle{\left|{\overrightarrow{\mu\:}}_{0f}\cdot\:{\widehat{\varepsilon\:}}_{\mathrm{d}}\right|}^{2}{\left(0\right){\Delta\:}}_{\mathrm{CPL}}\left(t\right)\rangle exp(-t/\tau\:)\end{aligned}$$

5 $$\begin{array}{l}\:{{\Delta\:}}_{\mathrm{CPL}}=\bigg[{\left|{\widehat{\varepsilon\:}}_{-}\cdot\:{\overrightarrow{\mu\:}}_{\mathrm{L}}+\left({\widehat{\varepsilon\:}}_{{\mathrm{Z}}_{\mathrm{L}}}\times\:{\widehat{\varepsilon\:}}_{-}\right)\cdot\:{\overrightarrow{m}}_{\mathrm{L}}\right|}^{2}\\-{\left|{\widehat{\varepsilon\:}}_{+}\cdot\:{\overrightarrow{\mu\:}}_{\mathrm{L}}+\left({\widehat{\varepsilon\:}}_{{\mathrm{Z}}_{\mathrm{L}}}\times\:{\widehat{\varepsilon\:}}_{+}\right)\cdot\:{\overrightarrow{m}}_{\mathrm{L}}\right|}^{2}\bigg]\end{array}$$

Below, the denominator of the emission dissymmetry factor corresponds to the depolarised (d) emission as given by

6 $$\:{I}_{\mathrm{d}}\left(\lambda\:,t\right)=\:{f}_{\mathrm{TL}}\left(\lambda\:,t\right)\langle {\left|{\overrightarrow{\mu\:}}_{0f}\cdot\:{\widehat{\varepsilon\:}}_{\mathrm{d}}\right|}^{2}{\left(0\right)\left|{\overrightarrow{\mu\:}}_{0f}\cdot\:{\widehat{\varepsilon\:}}_{\mathrm{d}}\right|}^{2}\left(t\right)\rangle exp(-t/\tau\:)$$

Throughout, the bracket notation, $\:\langle \dots\:\rangle$, stands for an average of orientation and time-correlation functions. The left- and right-hand polarisations are unit vectors $\:{\widehat{\varepsilon\:}}_{-}=\frac{1}{\sqrt{2}}\left({\varepsilon\:}_{{\mathrm{X}}_{\mathrm{L}}}-i{\varepsilon\:}_{{\mathrm{Y}}_{\mathrm{L}}}\right)$, $\:{\widehat{\varepsilon\:}}_{+}=\frac{1}{\sqrt{2}}\left({\varepsilon\:}_{{\mathrm{X}}_{\mathrm{L}}}+i{\varepsilon\:}_{{\mathrm{Y}}_{\mathrm{L}}}\right),\:$respectively, whereas $\:{\widehat{\varepsilon\:}}_{\mathrm{d}}=\frac{1}{\sqrt{2}}\left({\varepsilon\:}_{{\mathrm{X}}_{\mathrm{L}}}+{\varepsilon\:}_{{\mathrm{Y}}_{\mathrm{L}}}\right)\:$denotes the depolarised excitation. $\:{f}_{\mathrm{T}\mathrm{L}}\left(\lambda\:\right)\:$is a normalised line-shape function for the total emission which may, or may not be the same as $\:{f}_{\mathrm{C}\mathrm{P}\mathrm{L}}\left(\lambda\:\right)$ [3].

The CPL emission is detected along the laboratory fixed $\:{\mathrm{Z}}_{\mathrm{L}}$-axis, while the electric and magnetic transition dipoles are, by orientation, transformed into the laboratory $\:{\mathrm{X}}_{\mathrm{L}}{\mathrm{Y}}_{\mathrm{L}}$-plane (cf., Fig. 1). From Eq. 5, it follows that

7 $$\:{{\Delta\:}}_{\mathrm{C}\mathrm{P}\mathrm{L}}=\mathrm{I}\mathrm{m}\left\{{\mu\:}_{{\mathrm{X}}_{\mathrm{L}}}{m}_{{\mathrm{X}}_{\mathrm{L}}}+{\mu\:}_{{\mathrm{Y}}_{\mathrm{L}}}{m}_{{\mathrm{Y}}_{\mathrm{L}}}\right\}$$

Since $\:\overrightarrow{m}$ is a complex property, the imaginary ($\:\mathrm{I}\mathrm{m}$) part is evaluated.

Throughout this work the excitation radiation propagates along or perpendicular the direction of CPL detection (*vide infra*). Most often studies of the dissymmetry factor make use of excitation at various directions [2, 3] with respect to macroscopically isotropic systems. Here, to distinguish and to emphasise a potential interest in anisotropic systems, the $\:{\varGamma\:}_{\mathrm{C}\mathrm{P}\mathrm{L}}\left(\lambda\:\right)$ ratio is introduced.

## Results

### Organisation of the Subsections I-III

The first system treated constitutes a two-dimensional uniaxial sample (**I**), schematically displayed in Fig. 2. Upon introduction of this case an overall formalism and notation is introduced, which is further applied in the theoretical treatment of the following systems. Moreover, general symmetry properties are pointed out for intrinsically anisotropic systems doped with any kind of uniaxially oriented luminophores.

Secondly, three-dimensional uniaxial anisotropic systems are considered (**II**), e.g., represented by macroscopically aligned non-chiral liquid crystals. The experimental approach in the context of the time-resolution of CPL is also outlined.

Thirdly, CPL on macroscopically isotropic, but microscopically anisotropic systems, have been examined (**III**). Finally, linear polarised luminescence (LPL) experiments are proposed and considered.

#### I. Two-Dimensional Uniaxial Systems

Macroscopically uniaxial systems are associated with a laboratory frame ($\:{\mathrm{X}}_{\mathrm{L}},{\mathrm{Y}}_{\mathrm{L}},\:{\mathrm{Z}}_{\mathrm{L}}$), as is illustrated in Fig. 2. The depolarised (d) excitation beam propagates along the laboratory $\:{\mathrm{Z}}_{\mathrm{L}}$-axis and the CPL-emission is detected along the same axis. In response to a short excitation pulse, the time-dependence of $\:{{\updelta\:}I}_{\mathrm{d}}$ follows from Eqs. 3 and 7, according to,

8 $$\begin{array}{l}\:{{\updelta\:}I}_{\mathrm{d}}\left(t\right)\propto\:\langle \frac{1}{2}\left\{{{\mu\:}_{{\mathrm{X}}_{\mathrm{L}}}}^{2}+{{\mu\:}_{{\mathrm{Y}}_{\mathrm{L}}}}^{2}\right\}\left(0\right)\\\mathrm{I}\mathrm{m}\left\{{\mu\:}_{{\mathrm{X}}_{\mathrm{L}}}{m}_{{\mathrm{X}}_{\mathrm{L}}}+{\mu\:}_{{\mathrm{Y}}_{\mathrm{L}}}{m}_{{\mathrm{Y}}_{\mathrm{L}}}\right\}\left(t\right)\rangle exp(-t\:/\tau\:)\end{array}$$

Equation 8 is similar, and almost equal to the numerator of the emission dissymmetry factor (Eq. 1). Furthermore, $\:{{\updelta\:}I}_{\mathrm{d}}\left(t\right)$ is a time-correlation function mainly connected to the luminophore molecular reorientations. For an evaluation of Eq. 8, it is necessary to transform the orientation of the transition dipoles from a molecular (M) to the laboratory $\:\left\{\mathrm{L}=\left({{\mathrm{X}}_{\mathrm{L}}{,\mathrm{Y}}_{\mathrm{L}},\mathrm{Z}}_{\mathrm{L}}\right)\right\}$ frame. It is also necessary to account for their eventual reorientations, with respect to the laboratory frame, during the emission lifetime. The necessary transformations are most conveniently handled by means of first-rank irreducible tensor operators [5]. For this procedure, Eq. 8 needs to be rewritten^1^,

9 $$\begin{array}{l}\:{\delta\:I}_\mathrm{d}\left(t\right)\propto\\-\langle\begin{array}{c}\left\{{\mu\:}_{+1}\left(\mathrm{L}\right){\mu\:}_{-1}\left(\mathrm{L}\right)\right\}\left(0\right)\left\{-2\mathrm{i}\right\}\\\left\{{\mu\:}_{-1}\left(\mathrm{L}\right)m_{+1}\left(\mathrm{L}\right)+{\mu\:}_{+1}\left(\mathrm{L}\right)m_{-1}\left(\mathrm{L}\right)\right\}\left(t\right)\end{array}\:\rangle \\exp(-t\:/\tau\:)\end{array}$$

The average orientations, $\:\langle \dots\:..\rangle$, concern angular transformations ($\:{{\Omega\:}}_{\mathrm{M}\mathrm{L}}$) from a molecular (M) to a laboratory (L) coordinate system. The Eulerian transformation angles are $\:({{\alpha\:}_{\mathrm{M}\mathrm{L}}{,\beta\:}_{\mathrm{M}\mathrm{L}}{,\gamma\:}_{\mathrm{M}\mathrm{L}})\equiv\:{\Omega\:}}_{\mathrm{M}\mathrm{L}}$. In general, Eq. 9 depends on molecular reorientation motions, which are described by a conditional probability density accounted, denoted $\:G\left({{\Omega\:}}_{\mathrm{M}\mathrm{L}}\left(0\right)\left|{{\Omega\:}}_{\mathrm{M}\mathrm{L}}\left(t\right)\right.\right)$. The concept refers to the probability of an initially oriented molecule, $\:{{\Omega\:}}_{\mathrm{M}\mathrm{L}}\left(0\right)$, and its probable orientation at a time *t* later, $\:{{\Omega\:}}_{\mathrm{M}\mathrm{L}}\left(t\right)$. Hence, the total time-dependent orientation distribution density can be expressed by $\:f\left({{\Omega\:}}_{\mathrm{M}\mathrm{L}}\left(t\right)\right)=f\left({{\Omega\:}}_{\mathrm{M}\mathrm{L}}\left(0\right)\right)G\left({{\Omega\:}}_{\mathrm{M}\mathrm{L}}\left(0\right)\left|{{\Omega\:}}_{\mathrm{M}\mathrm{L}}\left(t\right)\right.\right)$. For an initially isotropic macroscopic distribution density, $\:f\left({{\Omega\:}}_{\mathrm{M}\mathrm{L}}\left(0\right)\right)=\:\frac{1}{8{\pi\:}^{2}}$, this value may represent a liquid, or a frozen liquid solution.

In order to evaluate the relations between experimental and molecular properties ($\:\overrightarrow{\mu\:}$ and $\:\overrightarrow{m}$), the transition dipoles are chosen with respect to a molecule fixed coordinate system (M), as is indicated in Fig. 2. The electric transition dipole coincides with the $\:{\mathrm{Z}}_{\mathrm{M}}$-axis, and the magnetic transition dipole is localised in the $\:{\mathrm{X}}_{\mathrm{M}}{\mathrm{Z}}_{\mathrm{M}}$-plane. According to the Appendix, the Eq. A1 by orientation transforms a tensor property from the molecular to the laboratory frame, ($\:{{\Omega\:}}_{\mathrm{M}\mathrm{L}}$).

The macroscopic and uniaxially ordered system is, for instance, an aligned liquid crystal, or several upon each other stacked lipid bilayers. This is doped with a single kind of enantiomeric luminophores. Due to interactions with the anisotropic environment, the luminophores form an uniaxial orientation distribution.

Since the orientation distribution density of the luminophores, *f*, is uniaxial with respect to the $\:{\mathrm{Z}}_{\mathrm{L}\:}$-axis, the distribution obeys symmetry criteria in accordance with,

10 $$\begin{array}{l}\:f({\alpha\:}_{\mathrm{M}\mathrm{L}},\:{\beta\:}_{\mathrm{M}\mathrm{L}},\:{\gamma\:}_{\mathrm{M}\mathrm{L}})\:=\\\:\frac{1}{2\pi\:\:}f({\beta\:}_{\mathrm{M}\mathrm{L}},\:{\gamma\:}_{\mathrm{M}\mathrm{L}})\:=\:\frac{1}{2\pi\:\:}f({\pi\:-\beta\:}_{\mathrm{M}\mathrm{L}},\:{2\pi\:-\gamma\:}_{\mathrm{M}\mathrm{L}})\end{array}$$

$\:f\left({{\Omega\:}}_{\mathrm{M}\mathrm{L}}\right)$ exhibits no dependence on$\:\:{\alpha\:}_{\mathrm{M}\mathrm{L}}$, which implies an isotropic rotation distribution about the $\:{\mathrm{Z}}_{\mathrm{L}\:}$-axis. The angular dependence of $\:f({\beta\:}_{\mathrm{M}\mathrm{L}},{\gamma\:}_{\mathrm{M}\mathrm{L}})$ follows from the mirror symmetry of the $\:{\mathrm{X}}_{\mathrm{L}}{\mathrm{Y}}_{\mathrm{L}}$-plane.

From Eq. 9 and Eqs. A1 and A2, it is a rather straightforward task to show that,

11 $$\begin{array}{l}\:{{\updelta\:}I}_{\mathrm{d}}\left(t\right)\propto\:{4\mu\:}_{0}^{3}\mathrm{I}\mathrm{m}{\:m}_{0}\langle \left\{-\frac{1}{3}+{\frac{1}{3}D}_{00}^{\left(2\right)}\left({{\Omega\:}}_{0}\right)\right\}\\\left\{-\frac{1}{3}+{\frac{1}{3}D}_{00}^{\left(2\right)}\left({\Omega\:}\right)\right\}-\frac{4}{\sqrt{3}}{{\mu\:}_{0}^{3}\:(m}_{+1}+{m}_{-1})\\\left\{-\frac{1}{3}+{\frac{1}{3}D}_{00}^{\left(2\right)}\left({{\Omega\:}}_{0}\right)\right\}\left\{{D}_{0+1}^{\left(2\right)}\left({\Omega\:}\right)\right\}\rangle exp(-t\:/\tau\:)\end{array}$$

Two important and extreme conditions of Eq. 11 need an examination. Firstly, the initial excited distribution density at the time $\:t=0$, $\:f\left({{\Omega\:}}_{0}\right)$, and secondly its value at times $\:t>$ $\:{t}_{\infty\:},$ that is when the excited molecules have reached an orientation equilibrium, $\:f\left({{\Omega\:}}_{\infty\:}\right)$, or differently expressed, their orientation has become uncorrelated with the initial excitation distribution. For negligible orientation correlation between excited molecules at $\:{t}_{\infty\:}$, and that prior to the excitation, it is reasonable to assume that $\:f\left({{\Omega\:}}_{\infty\:}\right)=f\left({{\Omega\:}}_{0}\right)$. This condition is hereafter referred to as the non-correlated (n) case. Furthermore, the initial condition ($\:t=0$) can be considered as that of a frozen (f) system, that is, a negligible reorientation of the luminophores on the emission timescale, implying that $\:f\left({{\Omega\:}}_{0}\right)=f\left({{\Omega\:}}_{\infty\:}\right).$.

For frozen uniaxially symmetric systems, the evaluation of Eq. 11 gives,

12 $$\begin{array}{l}\:{{\updelta\:}I}_{\mathrm{d}\mathrm{f}}\left(0\right)={{\updelta\:}I}_{\mathrm{d}\mathrm{f}}\left(t\right)\propto\:\:{\frac{4}{9}\mu\:}_{0}^{3}\mathrm{I}\mathrm{m}{\:m}_{0}\\\left[\frac{6}{5}-\frac{12}{7}\langle {D}_{00}^{\left(2\right)}\left({{\Omega\:}}_{0}\right)\rangle +\frac{18}{35}\langle {D}_{00}^{\left(4\right)}\left({{\Omega\:}}_{0}\right)\rangle \right]exp(-t\:/\tau\:)\end{array}$$

while for the non-correlated case, Eq. 11 reads,

13 $$\begin{array}{l}\:{{\updelta\:}I}_{\mathrm{d}\mathrm{n}}\left(t\right)\propto\:\:{\frac{4}{9}\mu\:}_{0}^{3}\mathrm{I}\mathrm{m}\:{m}_{0}\\\left[\left(1-2\langle {D}_{00}^{\left(2\right)}\left({{\Omega\:}}_{0}\right)\rangle +{\langle {D}_{00}^{\left(2\right)}\left({{\Omega\:}}_{0}\right)\rangle }^{2}\right)\right]exp(-t\:/\tau\:)\end{array}$$

The orientation averaged matrix elements ($\:\langle {D}_{00}^{\left(\mathrm{k}\right)}\left({{\Omega\:}}_{0}\right)\rangle$) in Eqs. 12 and 13 are referred to as order parameters [6] for which explicit trigonometric expressions are given by Eq. A3. Given a perfect orientation of the electric transition dipoles parallel to the $\:{\mathrm{Z}}_{\mathrm{L}\:}$-axis, the order parameters $\:\langle {D}_{00}^{\left(2\right)}\left({{\Omega\:}}_{0}\right)\rangle$ and $\:\langle {D}_{00}^{\left(4\right)}\left({{\Omega\:}}_{0}\right)\rangle$ are equal to one, while for the perfect perpendicular orientation corresponding values are -$\:\:\frac{1}{2}$ and $\:\frac{3}{8}$, respectively. Because of the uniaxial symmetry of $\:f\left({\beta\:}_{\mathrm{M}\mathrm{L}}\right)$, the order parameters $\:\langle {D}_{0\pm\:1}^{\left(\mathrm{k}\right)}\left({{\Omega\:}}_{0}\right)\rangle$ in Eq. 11 vanish. By the transformation the $\:{\mu\:}_{0}$- and $\:{m}_{0}$-tensor components into Cartesian coordinates, $\:{{\:\mathrm{I}\mathrm{m}\mu\:}_{0}m}_{0}=\left|\overrightarrow{\mu\:}\right|{m}_{{\mathrm{Z}}_{\mathrm{M}}}$= $\:\left|\overrightarrow{\mu\:}\right|\left|\overrightarrow{m}\right|cos\theta\:$, where $\:\theta\:$ denotes the angle between the inherent molecular directions of the electric and magnetic transition dipoles. In switching between two enantiomers of a luminophore, $\:\theta\:$ will change into $\:\pi\:-\theta\:$, which implies that the sign of the circular polarisation is also switched.

### How Does the Luminophore Orientation Influence CPL?

The key-questions concern to what extent and how circular polarised luminescence ($\:{{\updelta\:}I}_{\mathrm{d}}$) quantitatively differ between macroscopically isotropic or random systems (i), and macroscopically uniaxial systems? For this reason, frozen (f), as well as non-correlated (n) systems are considered in the following. For macroscopic isotropic (i) systems, $\:{{\updelta\:}I}_{\mathrm{d}\mathrm{f}\mathrm{i}\:}\propto\:\frac{4}{9}\mathrm{I}\mathrm{m}{\:\mu\:}_{0}{m}_{0}\left[\frac{6}{5}\right]\:$and $\:{{\updelta\:}I}_{\mathrm{d}\mathrm{n}\mathrm{i}\:}\propto\:\frac{4}{9}\mathrm{I}\mathrm{m}{\:\mu\:}_{0}{m}_{0}\left[1\right]$, which follows from Eqs. 12 and 13, respectively. For macroscopically anisotropic systems, $\:{{\updelta\:}I}_{\mathrm{d}\mathrm{f}\:}\:\mathrm{a}\mathrm{n}\mathrm{d}\:{\:{\updelta\:}I}_{\mathrm{d}\mathrm{n}}$ are $\:\propto\:\frac{4}{9}\mathrm{I}\mathrm{m}{\:\mu\:}_{0}{m}_{0}\:\mathrm{t}\mathrm{i}\mathrm{m}\mathrm{e}\mathrm{s}\:$factors, denoted by […], that depend on order parameters according to Eqs. 12 and 13. At the very extremes of perfect parallel or perpendicular orientation of the electric dipole with respect to the optic axis, the values of $\:\left[\dots\:\right]$ are 0 and $\:\frac{9}{4}$. Thus, the relative CPL increase is infinitely large for electric transition dipoles preferentially being oriented perpendicular the optical axis. To exemplify the influence a preferentially perpendicular orientation to the optic axis, the normalised distribution density function $\:f\left({\beta\:}_{\mathrm{M}\mathrm{L}}\right)=\frac{35}{32}{sin}^{6}{\beta\:}_{\mathrm{M}\mathrm{L}}$ has been assumed. $\:f\left({\beta\:}_{\mathrm{M}\mathrm{L}}\right)$ corresponds to a half-width of 50.6^o^, at its peak value. From this distribution density one obtains $\:\langle {D}_{00}^{\left(2\right)}\left({{\Omega\:}}_{0}\right)\rangle =\:-0.333$ and $\:\langle {D}_{00}^{\left(4\right)}\left({{\Omega\:}}_{0}\right)\rangle =0.223$, as well as, that $\:{{\updelta\:}I}_{\mathrm{d}\mathrm{f}\:}\propto\:\frac{4}{9}\mathrm{I}\mathrm{m}{\:\mu\:}_{0}{m}_{0}\left\{1.88\right\}\:$and $\:{{\updelta\:}I}_{\mathrm{d}\mathrm{n}\:}\propto\:\frac{4}{9}\mathrm{I}\mathrm{m}{\:\mu\:}_{0}{m}_{0}\left[1.78\right]$. The values are to be compared with isotropic non-correlated value of $\:{{\updelta\:}I}_{\mathrm{d}\mathrm{n}\mathrm{i}\:}\propto\:\frac{4}{9}\mathrm{I}\mathrm{m}{\:\mu\:}_{0}{m}_{0}\left[1.00\right]$. To summarise, the maximum degree of amplification is 225%.

Quite often CPL studies report on values of dissymmetric factors of various luminophores (cf [2, 3]).,. In order to calculate the dissymmetric factor, the experimental values of $\:{I}_{\mathrm{l}\mathrm{e}\mathrm{f}\mathrm{t}}\:\mathrm{a}\mathrm{n}\mathrm{d}\:{I}_{\mathrm{r}\mathrm{i}\mathrm{g}\mathrm{h}\mathrm{t}}$ are needed for a macroscopically anisotropic system. Since, the electric dipole transitions are represents the major contribution to the emission intensity, for most organic luminophores, it is reasonable to assume that,

14 $$\begin{array}{l}\:\left\{{I}_{\mathrm{l}\mathrm{e}\mathrm{f}\mathrm{t}}\left(t\right)+{I}_{\mathrm{r}\mathrm{i}\mathrm{g}\mathrm{h}\mathrm{t}}\left(t\right)\right\}\frac{1}{2}\approx\:\:{I}_{\mathrm{d}}\left(t\right)\\\propto\:\langle {\left\{{\mu\:}_{+1}{\mu\:}_{-1}\right\}}_{0}{\left\{{\mu\:}_{+1}{\mu\:}_{-1}\right\}}_{t}\rangle exp(-t\:/\tau\:)\end{array}$$

For systems in the frozen (f) and the non-correlated (n) case, Eq. 14 is then given by

15 $$\:{I}_{\mathrm{d}\mathrm{f}}\left(t\right)\propto\:\:{\frac{2}{9}\mu\:}_{0}^{4}\left(\frac{6}{5}-\frac{12}{7}\langle {D}_{00}^{\left(2\right)}\left({{\Omega\:}}_{0}\right)\rangle ++\frac{18}{35}\langle {D}_{00}^{\left(4\right)}\left({{\Omega\:}}_{0}\right)\rangle \right)exp(-t\:/\tau\:)$$

and

16 $$\:{I}_{\mathrm{d}\mathrm{n}}\left(t\right)\propto\:\:{\frac{2}{9}\mu\:}_{0}^{4}{\left(1-\langle {D}_{00}^{\left(2\right)}\left({{\Omega\:}}_{0}\right)\rangle \right)}^{2}exp(-t\:/\tau\:),$$

respectively.

To summarise, the dissymmetry-like factor (i.e., Eq. 3) for the frozen system is obtained from Eqs. 12 and 15, as well as the corresponding value for the non-correlated system from Eqs. 13 and 16. Both circumstances imply that $\:\delta\:{I}_{\mathrm{C}\mathrm{P}\mathrm{L}}\:\:$= $\:\mathrm{I}\mathrm{m}{\:\mu\:}_{0}{m}_{0}=\:\left|\overrightarrow{\mu\:}\right|\left|\overrightarrow{m}\right|cos\theta\:$. Thus, as expected, the dissymmetry-like factor represents an *inherent* property of a particular chiral luminophore, which is conveniently determined from experiments performed on macroscopically isotropic systems. For the determination of $\:\delta\:{I}_{\mathrm{C}\mathrm{P}\mathrm{L}}$, however, the present analysis demonstrates, that macroscopically isotropic systems are not strictly needed. For the emission dissymmetric factor, as defined, a value equal to + 2 means that the emission corresponds to 100% left-CP, whereas the opposite sign implies a 100% right-CP emission.

### Luminophore Reorientation on the Emission Timescale?

In order to account for luminophore reorientation during the emission lifetime, a theoretical model is needed. Except for molecules undergoing a spherical-like reorientation, this is a complex task, even for anisotropic molecules undergoing reorienting motions in isotropic liquids [7]. The molecular reorientation and dynamics in anisotropic systems can be described by means of a strong collision model [8], whereby.

17 $$\begin{array}{l}\:f\left({{\Omega\:}}_{\mathrm{M}\mathrm{L}}\left(t\right)\right)= \:\left\{f\left({{\Omega\:}}_{\mathrm{M}\mathrm{L}}\left(0\right)\right)-f\left({{\Omega\:}}_{\mathrm{M}\mathrm{L}}\left({t}_{\infty\:}\right)\right)\right\}\\exp\left(-t/{\tau\:}_{\mathrm{c}}\right)+f\left({{\Omega\:}}_{\mathrm{M}\mathrm{L}}\left({t}_{\infty\:}\right)\right)\end{array}$$

Here, $\:{\tau\:}_{\mathrm{c}}$ denotes an *effective* rotation correlation time. Taken together, Eqs. 12–14 allow a modelling of the emission dissymmetry numerator $\:{{\updelta\:}I}_{\mathrm{d}}\:$for time-resolved, as well as steady-state (s) experimental conditions. From Eq. 17 it follows that

18 $$\:{{\updelta\:}I}_{\mathrm{d}}\left(t\right)=\:\left[\left\{{{\updelta\:}I}_{\mathrm{d}\mathrm{f}}-\:{{\updelta\:}I}_{\mathrm{d}\mathrm{n}}\right\}exp\left(-t/{\tau\:}_{\mathrm{c}}\right)+{{\updelta\:}I}_{\mathrm{d}\mathrm{n}}\right]exp(-t/\tau\:)$$

19 $$\:\stackrel{-}{{{\updelta\:}I}_{\mathrm{d}\mathrm{s}}}=\:\frac{{{\updelta\:}I}_{\mathrm{d}\mathrm{f}}\:-\:{{\updelta\:}I}_{\mathrm{d}\mathrm{n}}}{1+\frac{\tau\:}{{\tau\:}_{\mathrm{c}}}}+{{\updelta\:}I}_{\mathrm{d}\mathrm{n}}$$

From independent and standard time-resolved depolarisation [1] experiments, the emission lifetime and the rotation correlation time can be determined. Thus, it is possible to estimate the influence of the ratio $\:\tau\:/{\tau\:}_{\mathrm{c}}$. Provided $\:\tau\:/{\tau\:}_{\mathrm{c}}\gg\:1,$ the time-resolved and steady-state behaviour only depend on the non-correlated value, i.e., $\:{{\updelta\:}I}_{\mathrm{d}\mathrm{n}}$. At ratios of $\:\tau\:/{\tau\:}_{\mathrm{c}}\ll\:1$, $\:{{\updelta\:}I}_{\mathrm{d}\mathrm{n}}$ represents $\:{{\updelta\:}I}_{\mathrm{d}}\left(t\right)\:$and $\:\stackrel{-}{{{\updelta\:}I}_{\mathrm{d}\mathrm{s}}}$ of a frozen system, and could alternatively by expressed as $\:{{\updelta\:}I}_{\mathrm{d}}\left(0\right)$.

#### II. Three-Dimensional Uniaxial Systems

Let us recall the excitation and emission configuration illustrated in Fig. 1. Consider a linearly polarised beam propagating along the $\:{\mathrm{Y}}_{\mathrm{L}\:}$-axis with its polarisation directed along the $\:{\mathrm{Z}}_{\mathrm{L}\:}$-axis, and $\:{{\updelta\:}I}_{{\mathrm{Z}}_{\mathrm{L}\:}}\left(t\right)\:$is monitored along the $\:{\mathrm{Z}}_{\mathrm{L}}$-axis. Notice that the $\:{\mathrm{Z}}_{\mathrm{L}}$-excitation polarisation creates a uniform excitation distribution about the $\:{\mathrm{Z}}_{\mathrm{L}\:}$-axis, which actually corresponds to an inversion of the previously discussed depolarised excitation distribution (cf., Eq. 8).

Next, the luminescence intensity, $\:\sigma\:I\left(t\right)$, is defined as a linear combination of the two experiments, $\:{I}_{{\mathrm{Z}}_{\mathrm{L}\:}{\mathrm{Z}}_{\mathrm{L}\:}}\left(t\right)+2{I}_{{\mathrm{Z}}_{\mathrm{L}\:}{\mathrm{Y}}_{\mathrm{L}\:}}\left(t\right)\equiv\:\sigma\:I\left(t\right)$. $\:{I}_{{\mathrm{Z}}_{\mathrm{L}\:}{\mathrm{Z}}_{\mathrm{L}\:}}\left(t\right)$ denotes the luminescence intensity detected with its linear polarisation parallel to the $\:{\mathrm{Z}}_{\mathrm{L}}$-axis, while $\:{I}_{{\mathrm{Z}}_{\mathrm{L}\:}{\mathrm{Y}}_{\mathrm{L}\:}}\left(t\right)$ is the intensity detected for the polarisation parallel to the $\:{\mathrm{Y}}_{\mathrm{L}\:}$-axis. Within this approach, as well as the strong-collision model it the follows that,

$$\begin{array}{l}\:\frac{{{\updelta\:}I}_{{\mathrm{Z}}_{\mathrm{L}\:}}\left(t\right)}{{\upsigma\:}I\left(t\right)\:}=\\-\frac{2}{3}\frac{\mathrm{I}\mathrm{m}{\mu\:}_{0}{m}_{0}\left[2\rho\:\:exp(-t/{\tau\:}_{\mathrm{c}})-1-\langle {D}_{00}^{\left(2\right)}\left({{\Omega\:}}_{0}\right)\rangle +{2\langle {D}_{00}^{\left(2\right)}\left({{\Omega\:}}_{0}\right)\rangle }^{2}\right]exp(-t/\tau\:)}{{\mu\:}_{0}^{2}\left[1+2\langle {D}_{00}^{\left(2\right)}\left({{\Omega\:}}_{0}\right)\rangle \right]exp(-t/\tau\:)}\end{array}$$

Here,

20 $$\:\rho\:=\:\frac{1}{5}+\frac{2}{7}\langle {D}_{00}^{\left(2\right)}\left({{\Omega\:}}_{0}\right)\rangle -{\langle {D}_{00}^{\left(2\right)}\left({{\Omega\:}}_{0}\right)\rangle }^{2}+\frac{18}{35}\langle {D}_{00}^{\left(4\right)}\left({{\Omega\:}}_{0}\right)\rangle$$

The corresponding ratio obtained under the steady-state conditions of Eq. 20 is given by,

21 $$\:\frac{\stackrel{-}{{{\updelta\:}I}_{{\mathrm{Z}}_{\mathrm{L}\:}\left(t\right)}}}{\stackrel{-}{\sigma\:I\left(t\right)\:}}=-\frac{2}{3}\frac{\mathrm{I}\mathrm{m}{m}_{0}{\mu\:}_{0}\left[\frac{2\rho\:}{1+\frac{\tau\:}{{\tau\:}_{\mathrm{c}}}}\:-1-\langle {D}_{00}^{\left(2\right)}\left({{\Omega\:}}_{0}\right)\rangle \:+{2\langle {D}_{00}^{\left(2\right)}\left({{\Omega\:}}_{0}\right)\rangle }^{2}\right]}{{\mu\:}_{0}^{2}\left[2\langle {D}_{00}^{\left(2\right)}\left({{\Omega\:}}_{0}\right)\rangle +1\right]}$$

### Experimental Approach To and the Available Molecular Information

Next to always, the photo-elastic modulation (PEM) technique is applied in measurements of the emission intensity differential ($\:{{\Delta\:}I}_{\mathrm{C}\mathrm{P}\mathrm{L}}$). Typically, the modulation frequencies are in the order of 10^4^ s^−1^. That is, the time-resolution of $\:{{\updelta\:}I}_{\mathrm{d}}\left(t\right)$ would be in the microsecond region or slower, typically corresponding to phosphorescence relaxation times. Considering frozen and liquid-like samples, for which $\:\tau\:/{\tau\:}_{\mathrm{c}}\gg\:1$, $\:{{\updelta\:}I}_{\mathrm{d}\mathrm{f}}\left(t\right)=\:{{\updelta\:}I}_{\mathrm{d}\mathrm{n}}exp(-t/\tau\:$) ($\:cf.,\:\mathrm{e}\mathrm{q}.11$), whereas the expected steady-state values are $\:\stackrel{-}{{{\updelta\:}I}_{\mathrm{d}\mathrm{s}}}=\:\stackrel{-}{{{\updelta\:}I}_{\mathrm{d}\mathrm{n}}}\:$(cf., Eq. 19).

Frequently fluorescence relaxation occurs on the ns-time scale, and therefore far beyond the time-resolution of PEM detection. For samples of which $\:\tau\:/{\tau\:}_{\mathrm{c}}\approx\:1$, PEM experiments would then refer to the steady-state condition, i.e., $\:\stackrel{-}{{{\updelta\:}I}_{\mathrm{d}\mathrm{s}}}$ (cf., Eq. 19).

What is the expected influence of translational diffusion on CPL measurements? Since excited luminophores due to translational motions may diffuse out of the excitation/detection beam spot, the CPL emission should decrease. For instance, assuming an isotropic diffusion rate of 10^−10^ m^2^s^−1^, the root mean-square displacements for the emission lifetimes 10 ns, 1$\:\:{\upmu\:}$s, and 1 ms are approximately 2.5, 250, and 7500 Å, respectively^2^. Thus, depending on the exposed spot size examined, the rate of translational diffusion might influence $\:{\updelta\:}{I}_{\mathrm{C}\mathrm{P}\mathrm{L}}$, as well as, $\:{{\Delta\:}I}_{\mathrm{C}\mathrm{P}\mathrm{L}}$ experiments.

A different approach to the detection of CPL was published [9] which circumvents PEM technique. Instead, the emitted CPL radiation is transmitted through an achromatic quarter-wave plate prior to a separate analysis of the two resulting and mutually perpendicular linearly polarised components. This approach might extend the applicability of $\:{\updelta\:}{I}_{\mathrm{C}\mathrm{P}\mathrm{L}}\left(t\right)$ experiments to a higher time resolution.

#### III. Macroscopic Isotropic and Microscopic Anisotropic Systems

To exemplify, micelles, lipid vesicles [10] and globular proteins are well-known examples of such systems. Luminophores can be solubilised in the former structures, and covalently connected to, or intrinsically present in proteins. Unilamellar vesicles consist of a single lipid bilayer which is curved to form a spherical shell. Consequently, the luminophores exhibit an isotropic orientation with respect to a laboratory frame ($\:{\mathrm{X}}_{\mathrm{L}},\:{\mathrm{Y}}_{\mathrm{L}},\:{\mathrm{Z}}_{\mathrm{L}})$, whereas their local orientation is anisotropic with respect to a frame ($\:{\mathrm{X}}_{\mathrm{T}},\:{\mathrm{Y}}_{\mathrm{T}},\:{\mathrm{Z}}_{\mathrm{T}})$ connected to the lipid bilayer. A spherical aggregate may undergo isotropic reorientation during the emission lifetime. For a luminophore rigidly attached to a globular particle

22 $$\begin{array}{l}\:\frac{{\delta\:I}_{\mathrm{d}}\left(t\right)}{\sigma\:I\left(t\right)}=\\\frac{{\langle {\left\{-{\mu\:}_{+1}\left(\mathrm{L}\right){\mu\:}_{-1}\left(\mathrm{L}\right)\right\}}_{0}\left\{-2\mathrm{i}\right\}\left\{{\mu\:}_{-1}\left(\mathrm{L}\right){m}_{+1}\left(\mathrm{L}\right)+{\mu\:}_{+1}\left(\mathrm{L}\right){m}_{-1}\left(\mathrm{L}\right)\right\}\:\rangle }_{\mathrm{t}}exp(-\raisebox{1ex}{$t$}\!\left/\:\!\raisebox{-1ex}{$\tau\:$}\right.)}{\langle {\left\{{\mu\:}_{0}^{2}\left(\mathrm{L}\right)\right\}}_{0}{\left\{{\mu\:}_{0}^{2}\left(\mathrm{L}\right)-2{\mu\:}_{+1}\left(\mathrm{L}\right){\mu\:}_{-1}\left(\mathrm{L}\right)\right\}}_{\mathrm{t}}\rangle exp(-\raisebox{1ex}{$t$}\!\left/\:\!\raisebox{-1ex}{$\tau\:$}\right.)}\end{array}$$

where, $\:\sigma\:I\left(t\right)$ is defined in the previous subsection (II) on three-dimensional systems.

Two orientation transformations are needed to relate the molecular luminophore frame ($\:{\mathrm{X}}_{\mathrm{M}},\:{\mathrm{Y}}_{\mathrm{M}},\:{\mathrm{Z}}_{\mathrm{M}})$ to an experimental setup, by means of two consecutive transformations, i.e., $\:{{\Omega\:}}_{\mathrm{M}\mathrm{T}}$ followed by $\:{{\Omega\:}}_{\mathrm{T}\mathrm{L}}.\:$If the macroscopic anisotropic system reorients during the luminescence lifetime, this also needs consideration. For a spherical aggregate, the reorientation is described by the conditional probability ($\:G\left({{\Omega\:}}_{\mathrm{T}\mathrm{L}}^{0}|{{\Omega\:}}_{\mathrm{T}\mathrm{L}},t\right)$), valid for a macroscopically isotropic three-dimensional system;

23 $$\begin{array}{l}\:G\left({{\Omega\:}}_{\mathrm{T}\mathrm{L}}^{0}|{{\Omega\:}}_{\mathrm{T}\mathrm{L}},t\right)=\\\frac{1}{8{\pi\:}^{2}}\sum\:{D}_{00}^{\left(\mathrm{k}\right)}\left({{\Omega\:}}_{\mathrm{T}\mathrm{L}}^{0}\right){D}_{00}^{\left(\mathrm{k}\right)}\left({{\Omega\:}}_{\mathrm{T}\mathrm{L}}\right)\:exp\left\{-\mathrm{k}\left(\mathrm{k}+1\right)\right\}Dt\end{array}$$

Equation 23 is the solution to the diffusion equation of a spherical particle that undergoes Brownian rotation [7]. A rotational correlation time ($\:{\tau\:}_{rot}$) is, for second rank tensors ($\:\mathrm{k}=2$), related to the rotational diffusion coefficient (*D*) by $\:{\tau\:}_{\mathrm{r}}=1/6D$. For unilamellar lipid vesicles dispersed in water, with the radii 100Å and 1000 Å, $\:{\tau\:}_{\mathrm{r}}$ corresponds to the time range of micro- and milliseconds, respectively. Hence, luminophores exhibiting long-lived emitting states may provide tools for determining aggregate size.

How to relate the order and dynamics of luminophores that reside in the lipid bilayer of vesicles to the observables, $\:{{\updelta\:}I}_{\mathrm{d}}(t$) and $\:\sigma\:\mathrm{I}\left(t\right)$? The luminophores may undergo local reorientation at a rate $\:1/{\tau\:}_{c}$, as well as a global reorientation rate due to the rotational diffusion rate ($\:6D$) of the vesicles. Expressions for $\:{{\updelta\:}I}_{\mathrm{d}}(t$) and $\:\sigma\:\mathrm{I}\left(t\right)\:$were here derived by means of previously applied orientation transformations. Included are local reorientations, as well as the dynamics of local and global reorientation. It turns out that the ratio between the observables is,

$$\begin{array}{l}\frac{{{\updelta\:}I}_{\mathrm{d}}\left(t\right)}{\sigma\:\mathrm{I}\left(t\right)}=\frac{-4{\mathrm{I}\mathrm{m}\:m}_{0}{\mu\:}_{0}}{3{\mu\:}_{0}^{2}}\:\bigg[1+\frac{2}{9}\left\{{\:{\upkappa\:}\:\mathrm{exp}(-t/{\tau\:}_{\mathrm{c}})+\langle {D}_{00}^{\left(2\right)}\left({{\Omega\:}}_{\mathrm{M}\mathrm{T}}\right)\rangle }^{2}\right\}\\+\frac{4}{45}\left\{{\:{\upkappa\:}\:\mathrm{exp}(-t/{\tau\:}_{\mathrm{c}})+\langle {D}_{00}^{\left(2\right)}\left({{\Omega\:}}_{\mathrm{M}\mathrm{T}}\right)\rangle }^{2}\right\}\:\mathrm{exp}(-t/{\tau\:}_{\mathrm{r}})\bigg]\frac{exp(-t/\tau\:)}{exp(-t/\tau\:)},\end{array}$$

where

24 $$\:\kappa\:=\:\frac{1}{5}+\:\frac{2}{7}\langle {D}_{00}^{\left(2\right)}\left({{\Omega\:}}_{\mathrm{M}\mathrm{T}}\right)\rangle -{\langle {D}_{00}^{\left(2\right)}\left({{\Omega\:}}_{\mathrm{M}\mathrm{T}}\right)\rangle }^{2}+\frac{18}{35}\langle {D}_{00}^{\left(4\right)}\left({{\Omega\:}}_{\mathrm{M}\mathrm{T}}\right)\rangle$$

The order parameters refer to the molecular frame with respect to the normal of the lipid bilayer.

The corresponding steady-state ratio is,

25 $$\begin{array}{l}\frac{\stackrel{-}{{{\updelta\:}I}_{\mathrm{d}}\left(t\right)}}{\stackrel{-}{\sigma\:\mathrm{I}\left(t\right)}}=\frac{-4{\mathrm{I}\mathrm{m}\:m}_{0}{\mu\:}_{0}}{3{\mu\:}_{0}^{2}}\bigg[1+\frac{2}{9}\left\{\frac{{\upkappa\:}}{1+\frac{\tau\:}{{\tau\:}_{\mathrm{c}}}+\frac{\tau\:}{{\tau\:}_{\mathrm{r}}}}+\frac{{\langle {D}_{00}^{\left(2\right)}\left({{\Omega\:}}_{\mathrm{M}\mathrm{T}}\right)\rangle }^{2}}{1+\frac{\tau\:}{{\tau\:}_{\mathrm{r}}}}\right\}\\+\frac{4}{45}\left\{\frac{{\upkappa\:}}{1+\frac{\tau\:}{{\tau\:}_{\mathrm{c}}}+\frac{\tau\:}{{\tau\:}_{\mathrm{r}}}}+\frac{{\langle {D}_{00}^{\left(2\right)}\left({{\Omega\:}}_{\mathrm{M}\mathrm{T}}\right)\rangle }^{2}}{1+\frac{\tau\:}{{\tau\:}_{\mathrm{r}}}}\right\}\bigg]\end{array}$$

In the case of rapid local and very slow global reorientations, as compared to the luminescence rate (i.e., $\:{\tau\:}_{\mathrm{c}}\ll\:\tau\:\ll\:{\tau\:}_{\mathrm{r}}$) Eq. 24 and 25 are simplified as

26 $$\:\frac{\stackrel{-}{{{\updelta\:}I}_{\mathrm{d}}\left(t\right)}}{\stackrel{-}{\sigma\:\mathrm{I}\left(t\right)}}=\frac{{{\updelta\:}I}_{\mathrm{d}}\left(t\right)}{\sigma\:\mathrm{I}\left(t\right)}=\frac{-4{\mathrm{I}\mathrm{m}\:m}_{0}{\mu\:}_{0}}{3{\mu\:}_{0}^{2}}\left[1+\frac{14}{45}{\langle {D}_{00}^{\left(2\right)}\left({{\Omega\:}}_{\mathrm{M}\mathrm{T}}\right)\rangle }^{2}\right]$$

Thus, the value of $\:\left|\langle {D}_{00}^{2}\left({{\Omega\:}}_{0}\right)\rangle \right|$ can be calculated from the experimental data. It might seem remarkable to obtain luminophore orientation from a macroscopic isotropic system. The curious reader might ask whether a detection procedure similar to CPL, also provides the same molecular information about *non-chiral* luminophores residing in these, or similar systems. Let the proposal be referred to as linear polarised luminescence (LPL). By means of the principal CPL set-up, repeatably discussed above, the detection concerns monitoring the difference in linearly polarised emission intensity. This is achieved by inserting an achromatic quarter-wave plate prior to the PEM, or by analysing the detected PEM-signal at twice its operational frequency [3].

A suggested LPL experiment, is described with reference to Fig. 1. For this description, however, a new laboratory frame $\:\left({\mathrm{X}}_{\mathrm{L}}^\prime,{\mathrm{Y}}_{\mathrm{L}}^\prime,{\mathrm{Z}}_{\mathrm{L}}^\prime\:\right)$ is introduced so that, $\:{-\mathrm{X}}_{\mathrm{L}}\curvearrowright\:{\mathrm{X}}_{\mathrm{L}}^\prime$ $\:{\mathrm{Y}}_{\mathrm{L}}\curvearrowright\:{\mathrm{Z}}_{\mathrm{L}}^\prime$, and $\:{\mathrm{Z}}_{\mathrm{L}}\curvearrowright\:{\mathrm{Y}}_{\mathrm{L}}^\prime$. The linearly polarised excitation beam propagates along the $\:{\mathrm{Y}}_{\mathrm{L}}^\prime$-axis with the polarisation directed along the $\:{\mathrm{Z}}_{\mathrm{L}}^\prime$-axis, which enables measurements of $\:{\delta\:I}_{{\mathrm{L}\mathrm{P}\mathrm{L}}_{\:}}\left(t\right)$. Furthermore, the linear emission intensity difference between the $\:{\mathrm{Z}}_{\mathrm{L}}^\prime$- and $\:{\mathrm{X}}_{\mathrm{L}}^\prime$-polarisations is detected, i.e., $\:\sigma\:I\left(t\right)$. Analogous to the ratio $\:{\delta\:I}_{{\mathrm{d}}_{\:}}\left(t\right)/\sigma\:I\left(t\right)\:$in Eq. 22), the corresponding LPL ratio is defined as,

27 $$\:\frac{{\delta\:I}_{{\mathrm{L}\mathrm{P}\mathrm{L}}_{\:}}\left(t\right)}{\sigma\:I\left(t\right)}=\frac{\langle {\mu\:}_{{\mathrm{Z}}_{\mathrm{L}}^\prime}^{2}\left(0\right)\left\{{\mu\:}_{{\mathrm{Z}}_{\mathrm{L}}^\prime}^{2}-{\mu\:}_{{\mathrm{X}}_{\mathrm{L}}^\prime}^{2}\right\}\left(t\right)\rangle exp\left(-t/\tau\:\right)}{\langle {\mu\:}_{{\mathrm{Z}}_{\mathrm{L}}^\prime}^{2}\left(0\right)\frac{1}{3}\left\{{\mu\:}_{{\mathrm{Z}}_{\mathrm{L}}^\prime}^{2}+2{\mu\:}_{{\mathrm{X}}_{\mathrm{L}}^\prime}^{2}\right\}\left(t\right)\rangle exp\left(-t/\tau\:\right)}$$

The particular combination of linear polarisations, in the denominator, ensures the independence of an orientation correlation between the an initially excited chromophore and its emission at a time later [1].

By an analogous procedure to that above, the following relations were derived;

28 $$\begin{array}{l}\:\frac{{\delta\:I}_{{\mathrm{L}\mathrm{P}\mathrm{L}}_{\:}}\left(t\right)}{\sigma\:I\left(t\right)}=\\\frac{2}{5}\frac{\left[\left\{\kappa\:\:exp(-t/{\tau\:}_{\mathrm{c}})+{\langle {D}_{00}^{\left(2\right)}\left({{\Omega\:}}_{0}\right)\rangle }^{2}\right\}\right]exp(-(t/\tau\:+\:t/{\tau\:}_{\mathrm{r}})}{exp(-t/\tau\:)}\end{array}$$

29 $$\:\frac{\stackrel{-}{{\delta\:I}_{{\mathrm{L}\mathrm{P}\mathrm{L}}_{\:}}\left(t\right)}}{\stackrel{-}{\sigma\:I\left(t\right)}}=\frac{2}{5}\left[\frac{\kappa\:}{1+\:\frac{\tau\:}{{\tau\:}_{\mathrm{c}}}+\frac{\tau\:}{{\tau\:}_{\mathrm{r}}}}+\frac{{\langle {D}_{00}^{\left(2\right)}\left({{\Omega\:}}_{0}\right)\rangle }^{2}}{1+\frac{\tau\:}{{\tau\:}_{\mathrm{r}}}}\right]$$

In the time regime of a local fast reorientation ($\:\frac{\tau\:}{{\tau\:}_{\mathrm{c}}}\gg\:1)$ and slow tumbling of the vesicles $\:(6D\tau\:\ll\:1)$, the Eq. 28-29 provide the value of $\:\left|\langle {D}_{00}^{2}\left({{\Omega\:}}_{0}\right)\rangle \right|$, according to

30 $$\:\frac{{\delta\:I}_{{\mathrm{L}\mathrm{P}\mathrm{L}}_{\:}}\left(t\right)}{\sigma\:I\left(t\right)}=\frac{\stackrel{-}{{\delta\:I}_{{\mathrm{L}\mathrm{P}\mathrm{L}}_{\:}}\left(t\right)}}{\stackrel{-}{\sigma\:I\left(t\right)}}=\frac{2}{5}{\langle {D}_{00}^{\left(2\right)}\left({{\Omega\:}}_{0}\right)\rangle }^{2}$$

Notice, the information concerning order and dynamics of chiral luminophores is expected to be invariant to both CPL and LPL experiments.

## Concluding Remarks

The present work intends to emphasise the influence of orientation, and reorientation dynamics on chiral luminophores during their emission lifetime. For the quantitative investigation of CPL, these aspects could enable an increasing output of CP intensity, as well as to further provide molecular insight into the emission circular intensity differential, $\:{{\Delta\:}I}_{\mathrm{C}\mathrm{P}\mathrm{L}}$.

By a reasonable effort, the present analysis could underpin various conceivable experimental setups on luminophore systems, which are connected to a various information content about microscopic anisotropic and macroscopically isotropic/anisotropic systems, not least in studies related to the biomolecular sciences. CPL should provide a higher experimental sensitivity, as compared to common CD absorption experiments. In this context, of course, it is important to be aware of the technical limitations (electronic as well as optical) of the particular experimental equipment used [2].

A different choice to the depolarised excitation along the optic axis would be two different linearly polarised directions by excitation radiation that propagates perpendicular to the optic axis. The two polarisations are directed either parallel or perpendicular to the optic axis. Hereby the initially created excitation distribution of any uniaxial system (frozen or not) exhibits a uniaxially, or *biaxially* symmetric orientation distribution, respectively.

In principle molecular luminophore order and dynamics, as determined from CPL experiments, can be obtained from common emission depolarisation experiments. The CPL approach, however, provides independent studies of chiral luminophores.

For macroscopic anisotropic samples the influence of linear and circular optical birefringence remains as common complications in linear dichroism and circular dichroism absorption [4], as well as, in CPL [2] and LPL spectroscopy. These circumstances are difficult to unambiguously neglect [11]. The experimental approach described here emphasises the creation of uniaxial excitation and emission distributions which are propagating along the axis of CPL detection. This should enable controlling the influence of birefringence, which is expected to decrease upon decreasing the spot size of detection. Taken together, the present study suggests how to overcome some fundamental experimental obstacles.

## Data Availability

Here, Data presented refer to theoretical results based on analytical calculations. Thus, no new experimental data are presented.

## Appendix

The formalism with conventions and definitions given below refer to Brink and Satchler [5]. The generalized irreducible transition dipoles, A_q_, (i.e., $\:{\mu\:}_{0}$, $\:{\mu\:}_{\pm\:1}\:$and, $\:{m}_{0},\:{\:m}_{\pm\:1}$) are orientationally transformed according to,

A1 $$\:{\mathrm{A}}_{\mathrm{p}}\left(\mathrm{L}\right)=\sum\:_{-1}^{1}{\mathrm{A}}_{\mathrm{q}}\left(\mathrm{M}\right){D}_{\mathrm{p}\mathrm{q}}^{\left(1\right)*}\left({{\Omega\:}}_{\mathrm{M}\mathrm{L}}\right)$$

In Eq. A1, $\:{D}_{\mathrm{p}\mathrm{q}}^{\left(1\right)*}\left({{\Omega\:}}_{\mathrm{M}\mathrm{L}}\right)\:$denotes a first rank Wigner orientation transformation matric operator from the M to the L frame. The symmetry criteria as above stated on uniaxial systems (e.g., a lipid bilayer), imply restrictions on mutual relations between different matrix elements $\:{D}_{\mathrm{p}\mathrm{q}}^{\left(\mathrm{k}\right)*}\left({{\Omega\:}}_{\mathrm{M}\mathrm{L}}\right)$. In the present study, the involved rank k = 0, 1, or 2.

The matrix elements are given by

A2 $$\begin{array}{l}{D}_{\mathrm{p}\mathrm{q}}^{\left(\mathrm{k}\right)*}\left({{\Omega\:}}_{\mathrm{M}\mathrm{L}}\right)\\=\:{{{exp}\left(i{\mathrm{p}\alpha\:}_{\mathrm{M}\mathrm{L}}\right){d}_{\mathrm{p}\mathrm{q}}^{\left(\mathrm{k}\right)}\left({\beta\:}_{\mathrm{M}\mathrm{L}}\right){exp}\left(\mathrm{q}i{\gamma\:}_{\mathrm{M}\mathrm{L}}\right)=(-1)}^{p-q}D}_{-\mathrm{p}-\mathrm{q}}^{\left(\mathrm{k}\right)}\left({{\Omega\:}}_{\mathrm{M}\mathrm{L}}\right)\end{array}$$

Some relations between common Wigner rotational matrix elements and the Eulerian $\:\beta\:$-angle [12] are;

A3 $$\begin{array}{l}{d}_{00}^{\left(2\right)}\left(\beta\:\right)=\frac{1}{2}(3{cos}^{2}\beta\:-1),{d}_{0-1}^{\left(2\right)}\left(\beta\:\right)={-d}_{01}^{\left(2\right)}=\\-\sqrt{\frac{3}{2}}sin\beta\:cos\beta,{\:d}_{0-2}^{\left(2\right)}\left(\beta\:\right)={d}_{02}^{\left(2\right)}=\\-\sqrt{\frac{3}{8}}{sin}^{2}\beta\:{d}_{00}^{\left(4\right)}\left(\beta\:\right)=\frac{1}{8}\left(35{cos}^{4}\beta\:-30{cos}^{2}\beta\:+3\right),\\{d}_{0-1}^{\left(4\right)}\left(\beta\:\right)=-{d}_{01}^{\left(4\right)}\left(\beta\:\right)=-5\sqrt{\frac{3}{2}}sin\beta\:\left(7{cos}^{3}\beta\:-3cos\beta\:\right),\\\:{d}_{0-2}^{\left(4\right)}\left(\beta\:\right)={d}_{02}^{\left(4\right)}\left(\beta\:\right)=\:\frac{1}{4}\sqrt{\frac{5}{2}}{sin}^{2}\beta\:\left(7{cos}^{2}\beta\:-1\right)\end{array}$$

In forming pairwise products between two matrix elements $\:{D}_{\mathrm{p}\mathrm{q}}^{\left(1\right)}\left({{\Omega\:}}_{\mathrm{M}\mathrm{L}}\right)$ and $\:{D}_{\mathrm{r}\mathrm{s}}^{\left(1\right)}\left({{\Omega\:}}_{\mathrm{M}\mathrm{L}}\right),$ this contraction [5] is given by

A4 $$\begin{array}{l}{D}_{\mathrm{p}\mathrm{q}}^{\left(1\right)}\left({{\Omega\:}}_{\mathrm{M}\mathrm{L}}\right)\:\mathrm{are}\:\mathrm{listed}\:\mathrm{below}\,{D}_{\mathrm{r}\mathrm{s}}^{\left(1\right)}\left({{\Omega\:}}_{\mathrm{M}\mathrm{L}}\right)=\\\sum\:_{\mathrm{C}}(2\mathrm{C}+1)\left(\begin{array}{ccc}1&\:1&\:\mathrm{C}\\\:\mathrm{q}&\:\mathrm{r}&\:\mathrm{c}\end{array}\right)\left(\begin{array}{ccc}1&\:1&\:\mathrm{C}\\\:\mathrm{q}&\:\mathrm{s}&\:\mathrm{c}^\prime\end{array}\right)\\{D}_{\mathrm{c}\mathrm{c}^\prime}^{\left(\mathrm{C}\right)*}\left({{\Omega\:}}_{\mathrm{M}\mathrm{L}}\right)\end{array}$$

The brackets $$\:\left(\begin{array}{ccc}1&\:1&\:\mathrm{C}\\\:\mathrm{q}&\:\mathrm{s}&\:\mathrm{c}^\prime\end{array}\right)$$ in Eq. A4 denote so-called (3-j) symbols, which are tabulated elsewhere [13]. In this particular work, the rank is $\:0\le\:\mathrm{C}\le\:4$.

## Footnotes

1 The relations between the spherical tensor operators ($\:{\mathrm{A}}_{\mathrm{j}}$) of first rank and the Cartesian components ($\:{\mathrm{A}}_{\mathrm{x}},\:{\mathrm{A}}_{\mathrm{y}},\:{\mathrm{A}}_{\mathrm{z}}$) are: $\:{\mathrm{A}}_{0}=\:{\mathrm{A}}_{\mathrm{z}}\:;\:{\mathrm{A}}_{\pm\:1}=\:\mp\:\frac{1}{\sqrt{2}}({\mathrm{A}}_{\mathrm{x}}\pm\:\:\mathrm{i}{\mathrm{A}}_{\mathrm{y}})$ [5].

2 The diffusion constant of H_2_O is $\:{2.3\cdot\:10}^{-9}$ m^2^s^−1^ in water at 298 K.
